# Integration of ultrasound radiomics features and clinical factors: A nomogram model for identifying the Ki-67 status in patients with breast carcinoma

**DOI:** 10.3389/fonc.2022.979358

**Published:** 2022-10-05

**Authors:** Jiangfeng Wu, Qingqing Fang, Jincao Yao, Lifang Ge, Liyan Hu, Zhengping Wang, Guilong Jin

**Affiliations:** ^1^ Department of Ultrasound, Dongyang People’s Hospital, Dongyang, China; ^2^ Department of Ultrasound, Tianxiang East Hospital, Yiwu, China; ^3^ Department of Ultrasound, Zhejiang Cancer Hospital, Hangzhou, China

**Keywords:** breast carcinoma, Ki-67, ultrasound, radiomics, nomogram

## Abstract

**Objective:**

The aim of this study was to develop and validate an ultrasound-based radiomics nomogram model by integrating the clinical risk factors and radiomics score (Rad-Score) to predict the Ki-67 status in patients with breast carcinoma.

**Methods:**

Ultrasound images of 284 patients (196 high Ki-67 expression and 88 low Ki-67 expression) were retrospectively analyzed, of which 198 patients belonged to the training set and 86 patients to the test set. The region of interest of tumor was delineated, and the radiomics features were extracted. Radiomics features underwent dimensionality reduction analysis by using the independent sample t test and least absolute shrinkage and selection operator (LASSO) algorithm. The support vector machine (SVM), logistic regression (LR), decision tree (DT), random forest (RF), naive Bayes (NB) and XGBoost (XGB) machine learning classifiers were trained to establish prediction model based on the selected features. The classifier with the highest AUC value was selected to convert the output of the results into the Rad-Score and was regarded as Rad-Score model. In addition, the logistic regression method was used to integrate Rad-Score and clinical risk factors to generate the nomogram model. The leave group out cross-validation (LGOCV) method was performed 200 times to verify the reliability and stability of the nomogram model.

**Results:**

Six classifier models were established based on the 15 non-zero coefficient features. Among them, the LR classifier achieved the best performance in the test set, with the area under the receiver operating characteristic curve (AUC) value of 0.786, and was obtained as the Rad-Score model, while the XGB performed the worst (AUC, 0.615). In multivariate analysis, independent risk factor for high Ki-67 status was age (odds ratio [OR] = 0.97, p = 0.04). The nomogram model based on the age and Rad-Score had a slightly higher AUC than that of Rad-Score model (AUC, 0.808 vs. 0.798) in the test set, but no statistical difference (p = 0.144, DeLong test). The LGOCV yielded a median AUC of 0.793 in the test set.

**Conclusions:**

This study proposed a convenient, clinically useful ultrasound radiomics nomogram model that can be used for the preoperative individualized prediction of the Ki-67 status in patients with BC.

## Introduction

Breast carcinoma (BC) is the most commonly diagnosed carcinoma and the main cause of cancer-associated mortality among women all over the world (1). The Ki-67 protein has repeatedly been confirmed as a significant clinical indicator for BC diagnosis and clinical decision-making, which is a nuclear antigen detected in all phases of the cell cycle, with the exception of the G0 phase (2). The Ki-67 is a well-established marker of tumor aggressiveness and proliferative activity, in which a higher Ki-67 expression reliably indicates not only more aggressive growth but also a greater risk of poorer prognosis and recurrence of BC (3–5). Hence, early detection of Ki-67 expression level is significant to improve and personalize treatment in patients with BC.

Preoperative assessment of the Ki-67 status is mainly detected by immunohistochemistry (IHC), which requires tissue sample typically obtained by core needle biopsy, and routinely evaluated by visual assessment by a pathologist (2, 6, 7). Whereas, the assessment of Ki-67 status based on a needle biopsy sample might not be representative of the whole tumor because of the tumor heterogeneity and relatively small sample size. Furthermore, in many critical cases, Ki-67 assessment can be unavailable where core needle biopsy is infeasible. Hence, creating an alternative, noninvasive method for predicting the Ki-67 status in patients with BC is clinically desirable.

Radiomics involves the high-throughput extraction and analysis of a great number of quantitative imaging features from digital images and can be utilized to identify the relationships between such quantitative imaging features and underlying tissue information (8, 9). Compared with conventional imaging metrics, radiomics has shown improved predictive values of multi-parametric imaging features. In recent years, a number of studies have found that radiomics analysis can be utilized to distinguish benign and malignant tumors (10, 11), detect lymph node metastasis (12, 13), and determine tumor molecular subtype (14, 15).

Several studies have reported that radiomics analysis could be used to assess the Ki-67 expression. For example, in a prior study by Zhang et al. (16), a prediction model based on radiomics of apparent diffusion coefficient (ADC) maps was developed and validated, which suggested that the ADC-based radiomics model could effectively predict the Ki-67 status in patients with BC before surgery. Furthermore, a study by Tagliafico et al. (17) showed that quantitative radiomics imaging features of breast tumor extracted from digital breast tomosynthesis (DBT) images were associated with BC Ki-67 expression. However, DBT and magnetic resonance imaging (MRI) are limited by economic cost and/or equipment availability.

To the best of our knowledge, the studies on assessing the relationships between the ultrasound radiomics features and Ki-67 status are very few. Thus, we studied whether ultrasound radiomics could be utilized as a predictive biomarker for the identification of Ki-67 status, and the aim of this study was to develop and validate an ultrasound-based radiomics nomogram model by integrating the clinical risk factors and ultrasound radiomics score (Rad-Score) to predict the Ki-67 status in patients with BC.

## Materials and methods

The study was approved by our Institutional Ethics Committee and performed on the basis of the Helsinki Declaration, and patient informed consent requirement was waived due to the retrospective nature of this study.

### Patient selection

Between March 2019 and April 2021, a total of 284 BC patients who met the following inclusion and exclusion criteria were retrospectively included in our study.

The inclusion criteria were (a) patients with BC confirmed by surgical or biopsy pathology; (b) BC patients with single and mass-like breast tumor (facilitating the subsequent segmentation of breast tumors); and (c) ultrasound examinations were carried out within 1 week before surgery.

The exclusion criteria were (a) insufficient quality of ultrasound images for radiomics study because of artifacts, calcifications or cystic changes that might have an extreme effect on pixel values; (b) tumors larger than 50 mm in diameter (incompletely displayed in a single plane); (c) patients who underwent radiotherapy and/or chemotherapy before ultrasound examination; and (d) clinical characteristics and postoperative IHC were incomplete.

### Pathological assessment

IHC analyses were carried out to detect the expression levels of estrogen receptor (ER), progesterone receptor (PR), human epidermal growth factor receptor 2 (HER2), and Ki-67 in each patient with BC. The status of ER and PR was considered as positive, if greater than 1% of tumor cells revealing positively stained nuclei (18). For HER2 status identification, an IHC score 3+ of HER2 was considered as positive, while an IHC score 0 or 1+ of HER2 was considered as negative. An IHC score 2+ was considered as indetermination, and then the fluorescence *in situ* hybridization (FISH) was carried out to assess gene amplification, and HER2 was classified as positive if the ratio ≥2.0 (19). For Ki-67 status, tumors with greater than 14% positive nuclei were considered as high expression, while other cases were considered as low expression (20).

### Clinical and pathological characteristics

Clinical data such as age, tumor size, tumor location, ultrasound-reported lymph node metastasis and ultrasound equipment were obtained from patients’ medical records. Status of ER, PR and HER2, Ki-67 level, pathology-reported lymph node metastasis and histological type of lesion were obtained by reviewing patients’ pathology reports.

### Image acquisition and segmentation

Preoperative ultrasound scannings were carried out by two sonographers (more than 5 years’ experience in the breast ultrasound). All breasts of the patients were scanned using LOGIQ E9 ultrasound system with a 6-15L linear array probe and Siemens Acuson S2000 with a 6-18L linear array probe. The ultrasound images were stored as the format of Digital Imaging and Communications in Medicine. A sonographer with no information about the lesion’s histopathology selected the largest plane of each breast lesion and delineated a two dimensional region of interest (ROI) that covered the whole lesion by using ITK-SNAP software (open source software; http://www.itk-snap.org).

### Feature extraction

A total of 788 ultrasound radiomics features were extracted from each patient and divided into four categories: 14 two dimension shape-based features; 18 first-order statistics features; 22 gray-level co-occurrence matrix (GLCM) features, 16 gray-level run length matrix (GLRLM) features, 16 gray-level size zone matrix (GLSZM) features, 14 gray-level dependence matrix (GLDM) features; and 688 features derived from first-order, GLCM, GLRLM, GLSZM and GLDM features using wavelet filter images. The extraction of the radiomics features was performed using the “pyradiomics” package of Python (version 3.7.11).

### Evaluation of interclass correlation coefficient

The consistency of the extracted ultrasound radiomics features was evaluated by the interclass correlation coefficient (ICC). Two sonographers drew ROIs in the same 50 randomly selected lesions and extracted the radiomics features. Then, interobserver reproducibility was evaluated by ICC between the 788 radiomics features of the 50 randomly selected lesions. The analysis revealed an ICC of > 0.70, demonstrating a good consistency of these characteristics.

### Radiomics feature selection

All the radiomics features were normalized with z-score normalization in the training and test sets to ensure that the scale of feature value was uniform and improve the comparability between features, which realized the proportional scaling of the original data (21). The calculating formula is listed below:


Y=(X−M)/S

where X is the initial value of radiomics feature, and M and S are the mean and standard deviation values of X, respectively, and Y is the transformed feature value.

The patients were randomly divided into the training and test set according to the ratio of 7:3. In the training set, a 2-step feature selection method was employed to select the most effective radiomics features. First, Kolmogorov-Smirnov test was first performed to assess whether data were normally distributed. Levene’s test was used to assess the equality of variances, and the independent sample t test or Welch’s t test was used to identify differences of the variables between the high and low Ki-67 status in the training set. The radiomics features that showed no significant differences were excluded. Second, the remaining radiomics features were further dimensionally reduced by using the penalized logistic regression with a least absolute shrinkage and selection operator (LASSO) algorithm working by attempting to shrink some coefficients of the model and set others to zero. An optimal parameter (Lambda) was computed using a tenfold cross-validation method to prevent overfitting. Thus, features with a non-zero coefficient in the model with an optimal parameter for Lambda were regarded as the most representative features.

### Construction and validation of machine learning classifiers

Based on the non-zero coefficient radiomics features extracted from ultrasound images, six advanced machine learning classifiers consisting of decision tree (DT), random forest (RF), support vector machine (SVM), logistic regression (LR), naive Bayes (NB) and XGBoost were adopted to construct the prediction model in the training set. The classifier with the highest AUC value in the test set was selected to convert the output of the results into Rad-Score which indicated the relative risk of high Ki-67 status, and the classifier was regarded as Rad-Score model.

### Construction and validation of clinical and nomogram models

**Figure 1:**
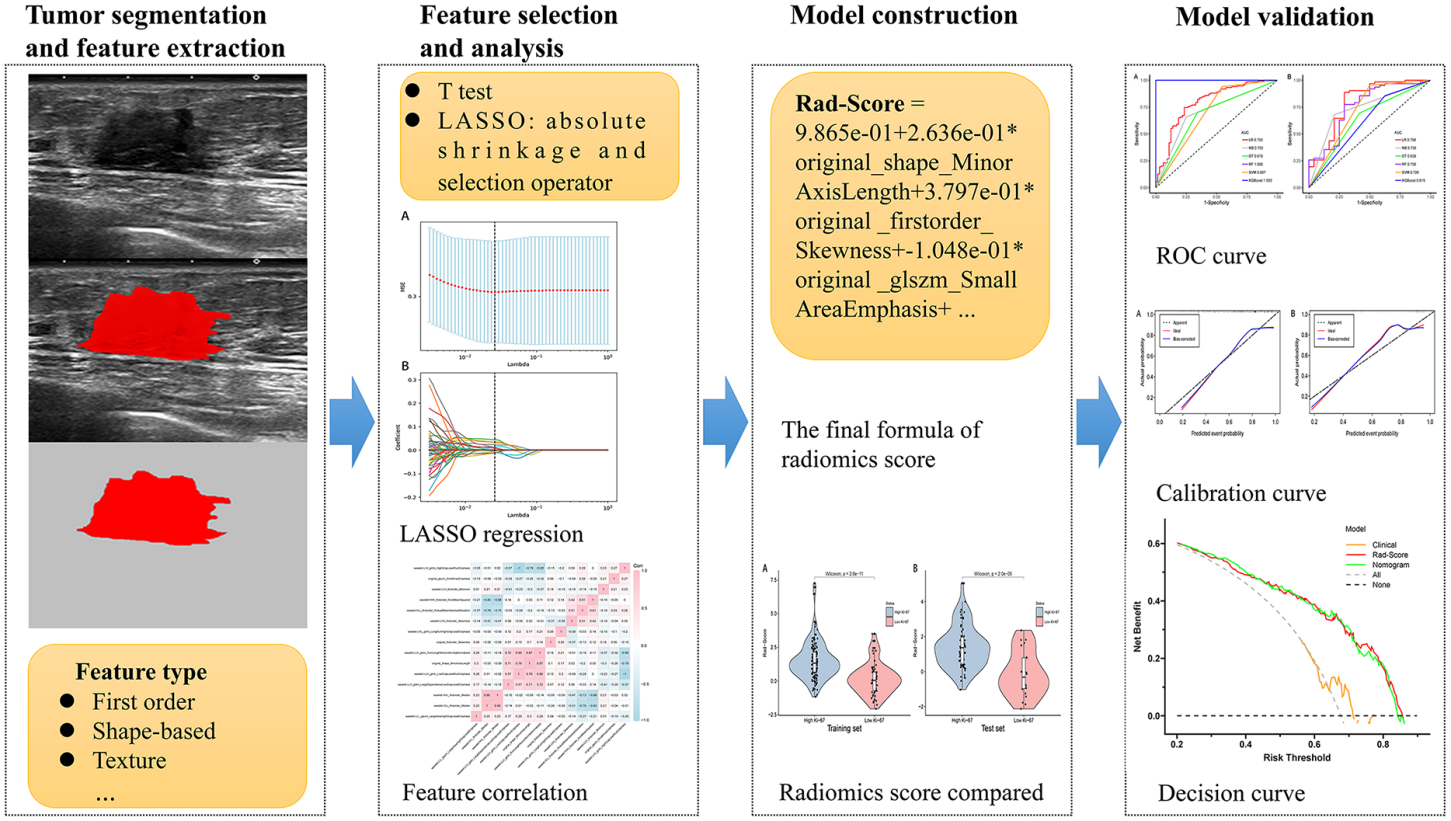
Flowchart of the processing step using the radiomics method for predicting the Ki-67 status. * means multiply.

In order to select clinical factors significantly related to high Ki-67 expression, univariate and multivariate logistic regression analyses were performed, and the clinical factors with p-value of < 0.05 were considered as risk factors. Meanwhile, logistic regression method was used to establish the clinical model based on the risk factors. Furthermore, for the aim of providing a personalized prediction model, the nomogram model combining Rad-Score and clinical risk factors was developed to predict high Ki-67 status. We evaluated the performance of each model in terms of sensitivity, specificity, positive predictive value (PPV), negative predictive value (NPV), accuracy, and the area under the receiver operating characteristic (ROC) curve (AUC). To verify the consistency of the nomogram model, the calibration curve (22) was plotted. Moreover, decision curve analysis (DCA) of the clinical model, Rad-Score model and nomogram model was implemented to obtain the model that maximized patient benefits (23). The flowchart of this research is shown in **Figure 1**.

### Statistical analysis

Statistical analyses were performed with the R software (version 3.5.1). The continuous variables with normal distribution and homogeneity of variance were shown as mean ± standard deviation (SD) and compared using independent sample t test, and otherwise were represented as the median (interquartile range) and compared by Mann-Whitney U test. The Fisher’s exact test or Chi-square test was used for comparing categorical variables. For all statistical tests, bilateral p< 0.05 was considered statistically significant.

## Results

### Clinical and pathological characteristics

**Figure 2:**
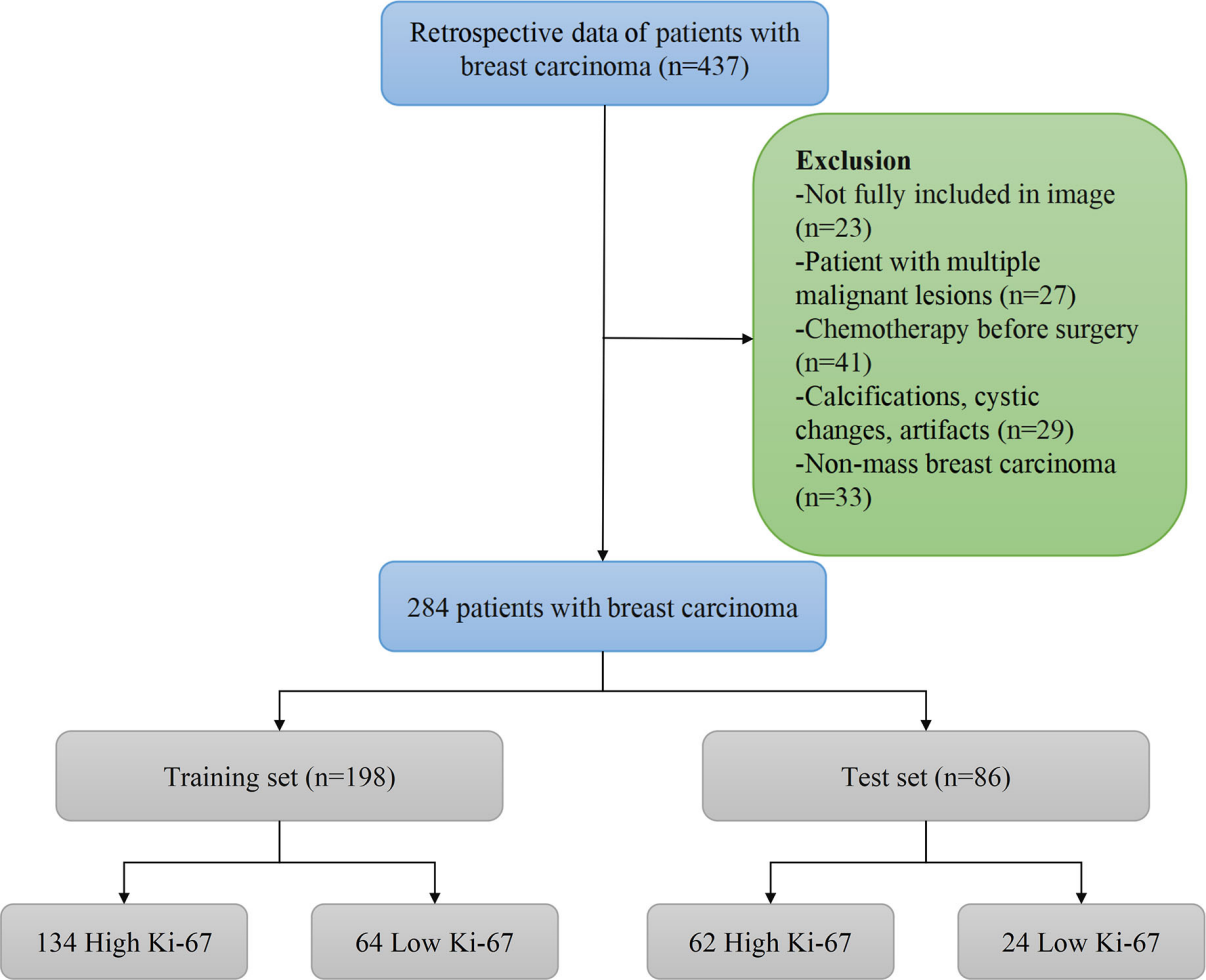
The patient enrollment process for this study.

On the basis of inclusion criteria, 437 patients were reviewed. Applying our exclusion criteria, a total of 284 patients were therefore included finally. Breast carcinomas were invasive ductal carcinoma in 228 patients, invasive lobular carcinoma in 27 patients, ductal carcinoma *in situ* in 17 patients, mucinous carcinoma in 8 patients, and papillary carcinoma in 4 patients. Among the 284 patients, we analyzed 198 patients in the training set and 86 patients in the test set. The training set included 134 and 64 patients with high and low Ki-67 expression, respectively, while the test set included 62 and 24 patients with high and low Ki-67 expression, respectively. The flowchart of patient selection process was revealed in **Figure 2**. The clinical and pathological characteristics of the training and test sets were compared, and there was no statistically significant difference found (p > 0.05) (**Table 1**). Furthermore, characteristics of patients in the high and low Ki-67 groups are listed in **Supplementary Table 1**.

**Table 1:** The baseline characteristics of the enrolled patients in the training and test sets.

Characteristic	Total set (n=284)	Training set (n=198)	Test set (n=86)	*p*-value
**Age** (year, mean ± SD)	53.65 ± 10.83	53.85 ± 11.28	53.17 ± 9.77	0.63
**Size** (mm, mean ± SD)	25.09 ± 11.21	25.08 ± 11.19	25.10 ± 11.33	0.99
**Location of disease**				
Right lobe	150	107	43	0.53
Left lobe	134	91	43	
**ER**				0.88
Positive	203	141	62	
Negative	81	57	24	
**PR**				0.88
Positive	167	117	50	
Negative	117	81	36	
**HER2**				0.98
Positive	73	51	22	
Negative	211	147	64	
**Histologic type**				0.74
Invasive ductal	228	160	68	
Other	56	38	18	
**Ultrasound equipment**				0.63
Siemens Acuson S2000	233	161	72	
LOGIQ E9	51	37	14	
**US-reported LN**				0.47
Metastasis positive	123	83	40	
Metastasis negative	161	115	46	
**Pathology-reported LN**				0.24
Metastasis positive	160	107	53	
Metastasis negative	124	91	33	
**Ki-67** (%, mean ± SD)	29.39 ± 22.96	28.04 ± 22.16	32.51 ± 24.57	0.13
**Radiomics score** (median, IQR)	0.90 (0.05, 1.74)	0.89 (0.06, 1.76)	0.96 (0.01, 1.72)	0.93

ER, estrogen receptor; PR, progesterone receptor; HER2, human epidermal growth factor receptor 2; SD, standard deviation; IQR, interquartile range; LN, lymph node; US, ultrasound.

### Radiomics feature extraction and selection

**Figure 3:**
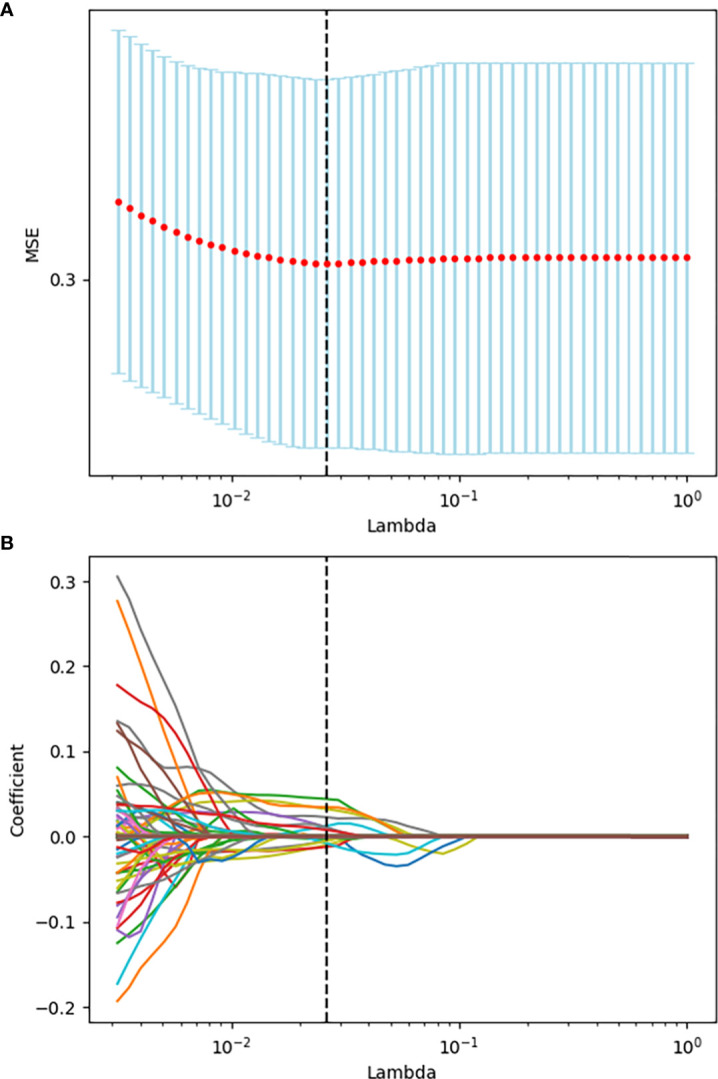
Tuning parameter selection using the LASSO regression in the training set. **(A)** The optimal penalization coefficient lambda was generated in the LASSO *via* tenfold cross-validation. The lambda value of the minimum mean square error for the training set was given for the features with non-zero selection coefficient; **(B)** LASSO coefficient profiles of the radiomics features.

**Figure 4:**
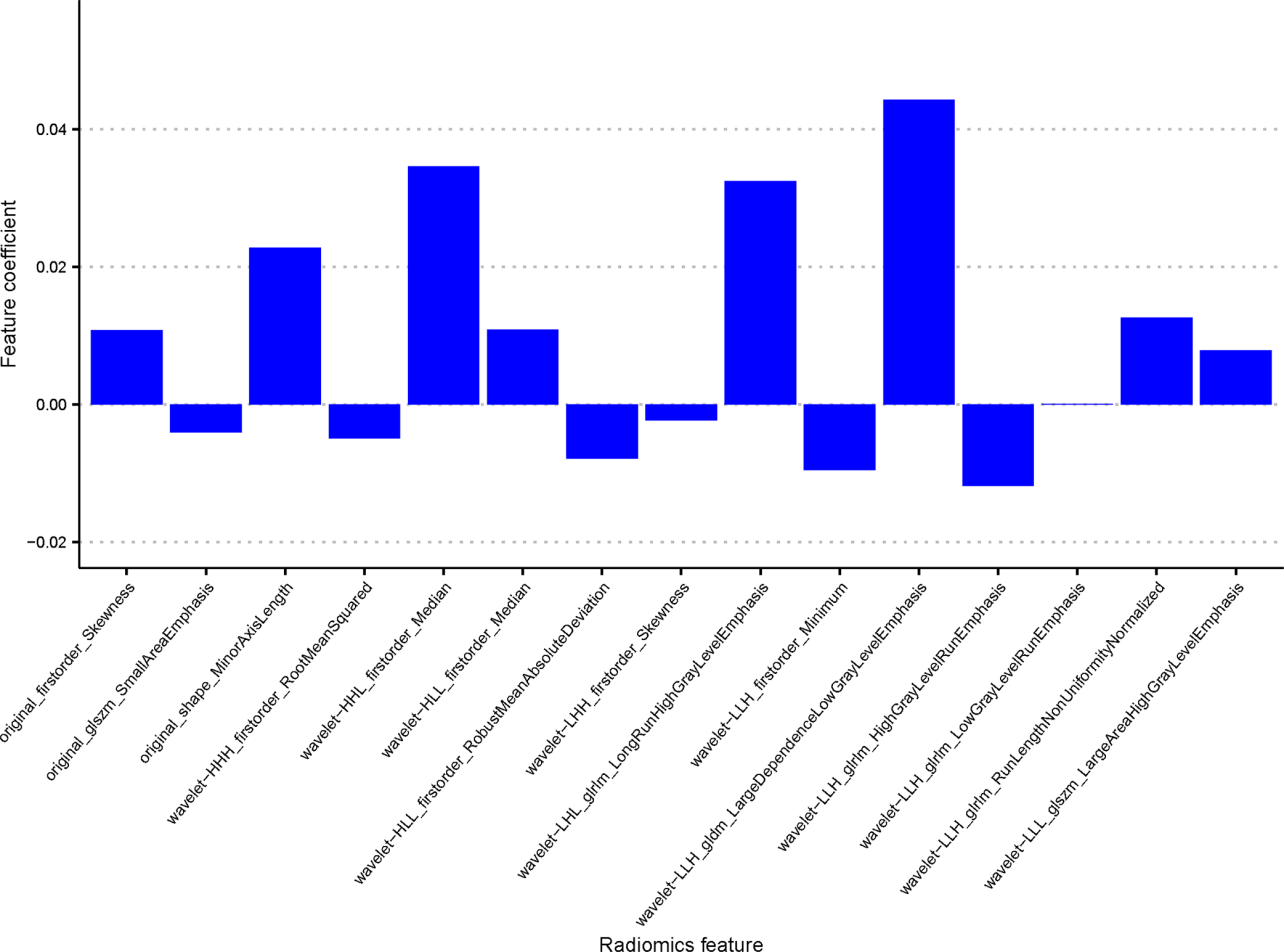
A non-zero coefficient profile plot of the 15 selected radiomics features derived from the LASSO regression was drawn.

**Figure 5:**
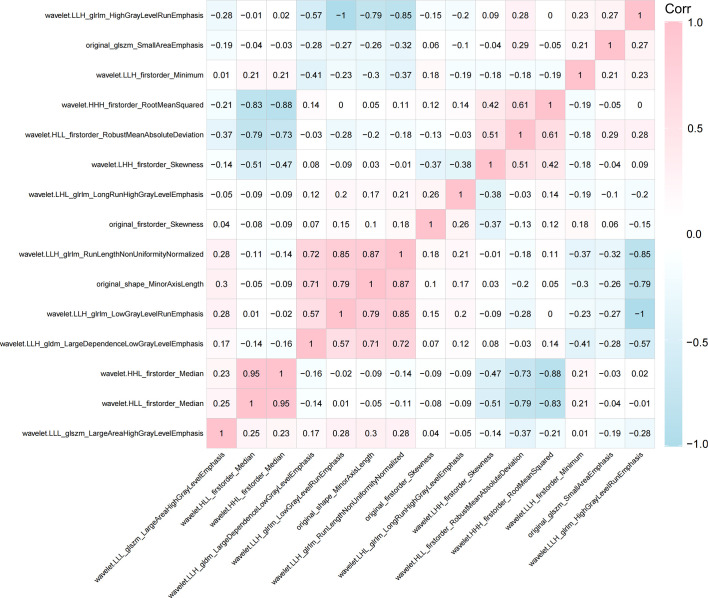
Pearson correlation coefficient heatmap of the selected features on predicting the high Ki-67 status. Red color denotes a positive correlation, blue color denotes a negative correlation, and the shade of the color indicates the correlation degree.

Seven hundred and eighty eight radiomics features were extracted from ultrasound image of each enrolled patient. The interobserver reproducibility of ultrasound radiomics features extracted between the two sonographers for 50 randomly selected lesions was good (ICC > 0.70). After evaluating the differences of radiomics features by using the independent sample t test, there were 336 features retained. Finally, the optimal Lambda (Lambda = 0.026203985288583486) was determined for the LASSO regression, and 15 features with non-zero coefficients were selected to predict the high Ki-67 expression of BC patients (**Figure 3**). Detailed information on these high Ki-67 expression-related features is available in **Table 2** and the weight coefficients of the selected features are shown in **Figure 4**. Furthermore, the Pearson correlation coefficient between any pair of selected features was computed, and the Pearson correlation coefficient matrix heatmap is revealed in **Figure 5**.

**Table 2:** List of the selected features with non-zero coefficients.

Image type	Feature class	Feature name
original	shape	MinorAxisLength
original	firstorder	Skewness
original	glszm	SmallAreaEmphasis
wavelet-LLH	firstorder	Minimum
wavelet-LLH	glrlm	HighGrayLevelRunEmphasis
wavelet-LLH	glrlm	LowGrayLevelRunEmphasis
wavelet-LLH	glrlm	RunLengthNonUniformityNormalized
wavelet-LLH	gldm	LargeDependenceLowGrayLevelEmphasis
wavelet-LHL	glrlm	LongRunHighGrayLevelEmphasis
wavelet-LHH	firstorder	Skewness
wavelet-HLL	firstorder	Median
wavelet-HLL	firstorder	RobustMeanAbsoluteDeviation
wavelet-HHL	firstorder	Median
wavelet-HHH	firstorder	RootMeanSquared
wavelet-LLL	glszm	LargeAreaHighGrayLevelEmphasis

### Machine learning classifier construction

On the basis of the 15 non-zero coefficient features, six machine learning classifiers (DT, RF, SVM, LR, NB and XGBoost) were then utilized to establish the prediction model. The sensitivity, specificity, accuracy, PPV, NPV, true positive (TP), false positive (FP), false negative (FN), true negative (TN), and AUC values of the six classifiers are shown in **Table 3**.

**Table 3:** Predictive performance of the six machine learning classifiers in the training and test sets.

Model	Set	AUC (95% CI)	SEN (%)	SPE (%)	ACC (%)	PPV (%)	NPV (%)	TP	FP	FN	TN
LR	Training	0.793 (0.722-0.863)	74.6%	76.6%	75.3 (%)	87.0%	59.0%	100	15	34	49
	Test	0.798 (0.679-0.918)	88.7%	70.8%	83.7%	88.7%	70.8%	55	7	7	17
SVM	Training	0.697 (0.632-0.761)	94.0%	45.3%	78.3%	78.3%	78.4%	126	35	8	29
	Test	0.726 (0.620-0.832)	95.2%	50.0%	82.6%	83.1%	80.0%	59	12	3	12
RF	Training	1.000 (1.000-1.000)	100.0%	100.0%	100.0%	100.0%	100.0%	134	0	0	64
	Test	0.756 (0.634-0.878)	77.4%	70.8%	75.6%	87.3%	54.8%	48	7	14	17
DT	Training	0.679 (0.609-0.749)	70.1%	65.6%	68.7%	81.0%	51.2%	94	22	40	42
	Test	0.638 (0.522-0.755)	69.4%	58.3%	66.3%	81.1%	42.4%	43	10	19	14
XGBoost	Training	1.000 (1.000-1.000)	100.0%	100.0%	100.0%	100.0%	100.0%	134	0	0	64
	Test	0.615 (0.507-0.723)	85.5%	37.5%	72.1%	77.9%	50.0%	53	15	9	9
NB	Training	0.703 (0.636-0.770)	65.7%	75.0%	68.7%	84.6%	51.1%	88	16	46	48
	Test	0.735 (0.633-0.836)	67.7%	79.2%	70.9%	89.4%	48.7%	42	5	20	19

DT, decision tree; RF, random forest; SVM, support vector machine; LR, logistic regression; NB, naive bayes; AUC, area under the curve; SEN, sensitivity; SPE, specificity; ACC, accuracy; PPV, positive predictive value; NPV, negative predictive value; TP, true positive; FP, false positive; FN, false negative; TN, true negative; CI, confidential interval.

**Figure 6:**
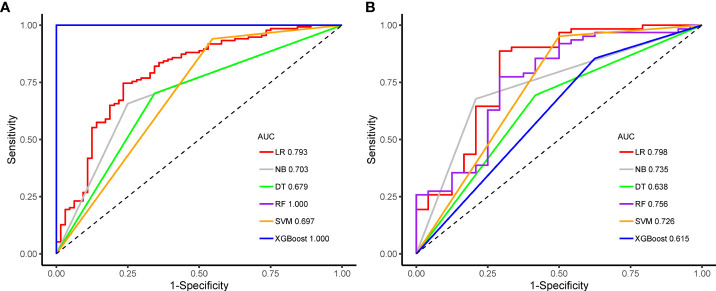
Receiver operating characteristic curves of the six machine learning classifiers predicting the high Ki-67 status in the training **(A)** and test sets **(B)**.

Among them, the XGBoost and RF classifiers were over-fitted, and had perfect discriminating ability in the training set but significantly reduced performance in the test set. The AUC values of the six machine learning classifiers ranged from 0.615 to 0.798 in the test set, with the LR classifier performing the best and XGBoost classifier performing the worst; the accuracy was between 66.3% in the DT classifier and 83.7% in the LR classifier. In the test set, the AUC values between the three classifiers of LR, SVM and NB were comparable (0.798 vs. 0.726 vs. 0.735), and no statistical differences were found by DeLong test. However, the LR classifier achieved the highest AUC value and was obtained as the Rad-Score model. A comparison of the ROC curves of the six machine learning classifiers in the training set and test set is shown in **Figure 6**. In addition, the AUC values between any pair of the classifiers were compared and the p values were calculated by DeLong test, which are revealed in **Supplementary Table 2**.

**Figure 7:**
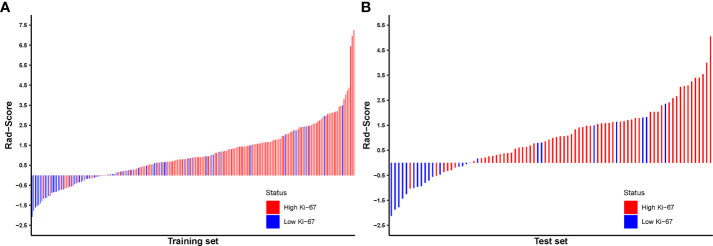
Radiomics score for each breast carcinoma patient in the training **(A)** and test sets **(B)**.

**Figure 8:**
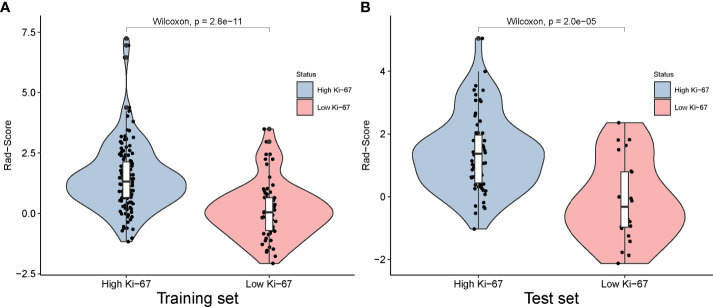
Distribution of radiomics score value of the high and low Ki-67 expression in the training and test sets.

The Rad-Score for each patient in the training and test sets was calculated based on the LR classifier for further analysis and is revealed in **Figure 7**. The corresponding fitting formula is listed in **Supplementary Material Data S1**. In the training set, the medians of Rad-Score were significant difference between the high and low Ki-67 groups (1.31 vs. 0.04, p< 0.001), and the same results were achieved in the test set (1.37 vs. -0.32, p< 0.001) in the test set (**Figure 8**; **Table 4**).

**Table 4:** Rad-Score for the training and test sets.

Rad-Score	High Ki-67 (median, IQR)	Low Ki-67 (median, IQR)	*p*-value
**Training set**	1.31 (0.65, 2.13)	0.04 (-0.71, 0.65)	<0.001
**Test set**	1.37 (0.44, 1.97)	-0.32 (-0.97, 0.79)	<0.001

IQR, interquartile range.

### Clinical model and nomogram model

**Figure 9:**
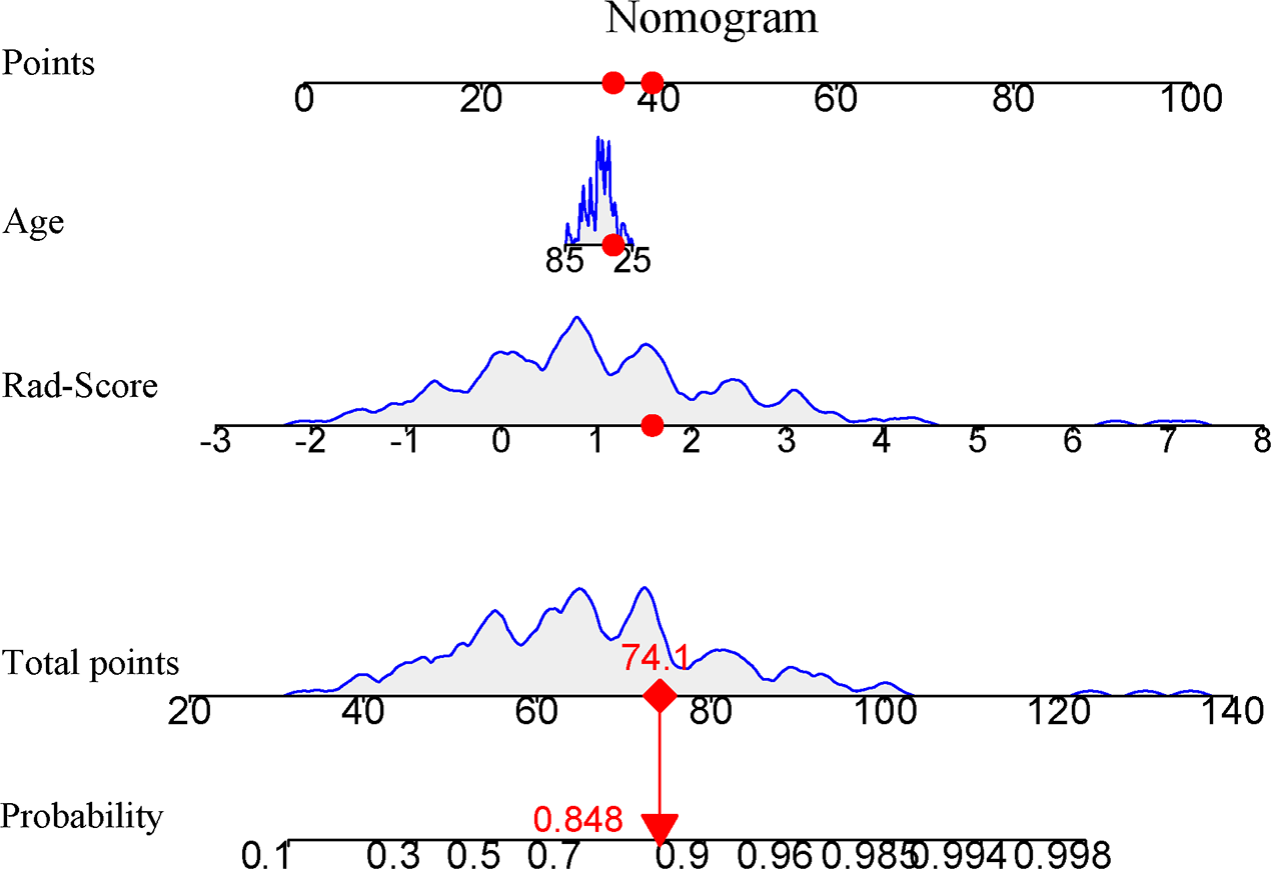
Nomogram based on the combination of the clinical risk factors and Rad-Score was developed using logistic regression analysis. If a patient with the radiomics score of 1.637 and age of 56, and then the probability of the high Ki-67 expression of breast carcinoma is 0.848 (red numbers).

The univariate and multivariate logistic regression analysis were applied to find independent predictors for the high Ki-67 status. The results are shown in **Table 5**, indicating that the age was the significant factor associated with the high Ki-67 expression. Then, the age as an independent predictor was adopted to develop the clinical model by using the logistic regression method. At the same time, based on the results of multivariate logistic regression analysis, the nomogram model was established by combining the age and Rad-Score (**Figure 9**).

**Table 5:** The results of logistic regression.

Clinical factors	Univariate logistic regression		Multivariable logistic regression	
	OR (95% CI)	*p*-value	OR (95% CI)	*p*-value
**Age**	0.97 (0.95, 0.99)	**0.02**	0.97 (0.95, 1)	**0.04**
**Location**	1.03 (0.62, 1.71)	0.90	NA	NA
**Size**	1.07 (1.04, 1.10)	**< 0.001**	NA	NA
**US equipment**	1.02 (0.53, 1.96)	0.95	NA	NA
**US-reported LN**	1.52 (0.90, 2.54)	0.11	NA	NA
**Rad-Score**	2.78 (2.08, 3.73)	**< 0.001**	2.75 (2.05, 3.67)	**< 0.001**

CI, confidence interval; NA, not applicable; LN, lymph node; US, ultrasound; OR, odds ratio. Bold values means statistical difference as P value < 0.05.

**Figure 10:**
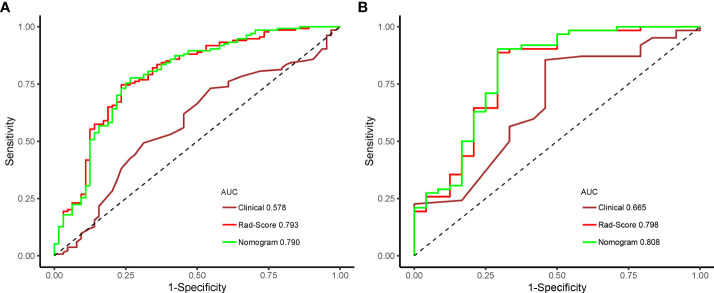
Receiver operating characteristic curves of the three models predicting the high Ki-67 expression in the training **(A)** and test sets **(B)**.

Furthermore, the performances of the clinical, Rad-Score, and nomogram models in the training set and test set were compared. As shown in **Table 6**, the nomogram model performed the best in the test set (AUC, 0.808), followed by the Rad-Score model (AUC, 0.798), while the clinical model performed the worst (AUC, 0.665). The AUC values were compared by the pairwise DeLong test, which indicated that in the test set, the AUC values of the nomogram model and the clinical model were significant statistical difference (AUC, 0.808 vs. 0.665; DeLong test, p = 0.04). Although there were differences in AUC values between the nomogram model and the Rad-Score model, there was no significant statistical difference (AUC, 0.808 vs. 0.798; DeLong test, p = 0.144). ROC curves of the three models to predict the Ki-67 status are shown in **Figure 10**.

**Table 6:** Predictive performances of the models predicting the Ki-67 status in patients with BC.

Model	Set	AUC	SEN (%)	SPE (%)	ACC (%)	PPV (%)	NPV (%)	TP	FP	FN	TN
Clinical	Training	0.578	73.1%	45.3%	64.1%	73.7%	44.6%	98	35	36	29
	Test	0.665	85.5%	54.2%	76.7%	82.8%	59.1%	53	11	9	13
Rad-Score	Training	0.793	74.6%	76.6%	75.3%	87.0%	59.0%	100	15	34	49
	Test	0.798	88.7%	70.8%	83.7%	88.7%	70.8%	55	7	7	17
Nomogram	Training	0.790	77.6%	73.4%	76.3%	86.0%	61.0%	104	17	30	47
	Test	0.808	90.3%	70.8%	84.9%	88.9%	73.9%	56	7	6	17

AUC, area under the curve; SEN, sensitivity; SPE, specificity; ACC, accuracy; PPV, positive predictive value; NPV, negative predictive value; Rad-Score, radiomics score; TP, true positive; FP, false positive; FN, false negative; TN, true negative.

The leave group out cross-validation (LGOCV) method was performed 200 times to verify the reliability and stability of the results, which yielded 200 AUC values ranging from 0.590 to 0.965 and a high median AUC (0.793 in the test set), indicating that the results of the nomogram model was reliable and stable (**Supplementary Figure 1**).

### Model performance evaluation

**Figure 11:**
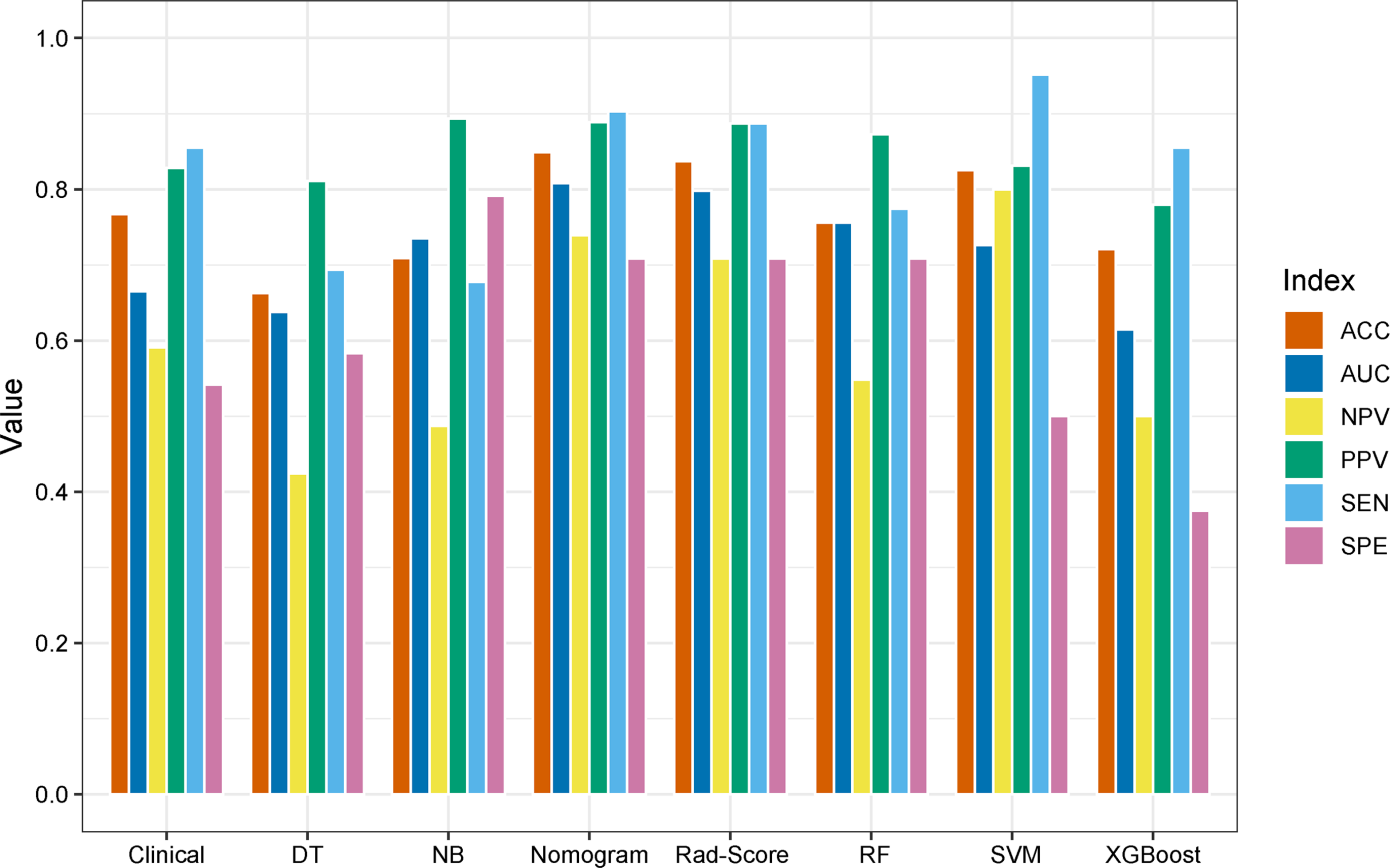
Bar plot of the performances of the eight prediction models in the test set.

The performance of eight models consisting of the six machine learning classifiers, clinical model and nomogram model in the test set is shown in **Figure 11**. The nomogram model has the highest AUC value (0.808) and accuracy (84.9%), SVM has the highest sensitivity (95.2%), and NB has the highest specificity (79.2%). To sum up, the overall discrimination performance of the nomogram model was better than that of the other models.

### Clinical application of prediction models

**Figure 12:**
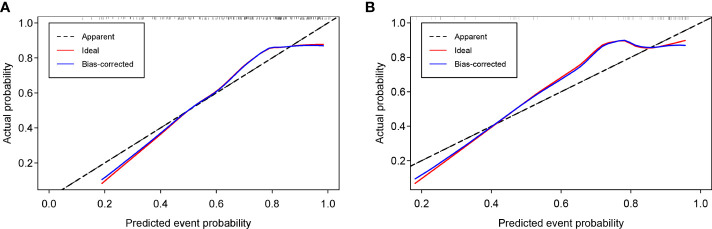
Calibration curves of the nomogram model in the training **(A)** and test sets **(B)**.

The calibration curves for the nomogram model were tested using Hosmer-Lemeshow test, and yielded nonsignificant results due to both p values > 0.05 in the training and test sets, providing evidence of good calibration (**Figure 12**).

**Figure 13:**
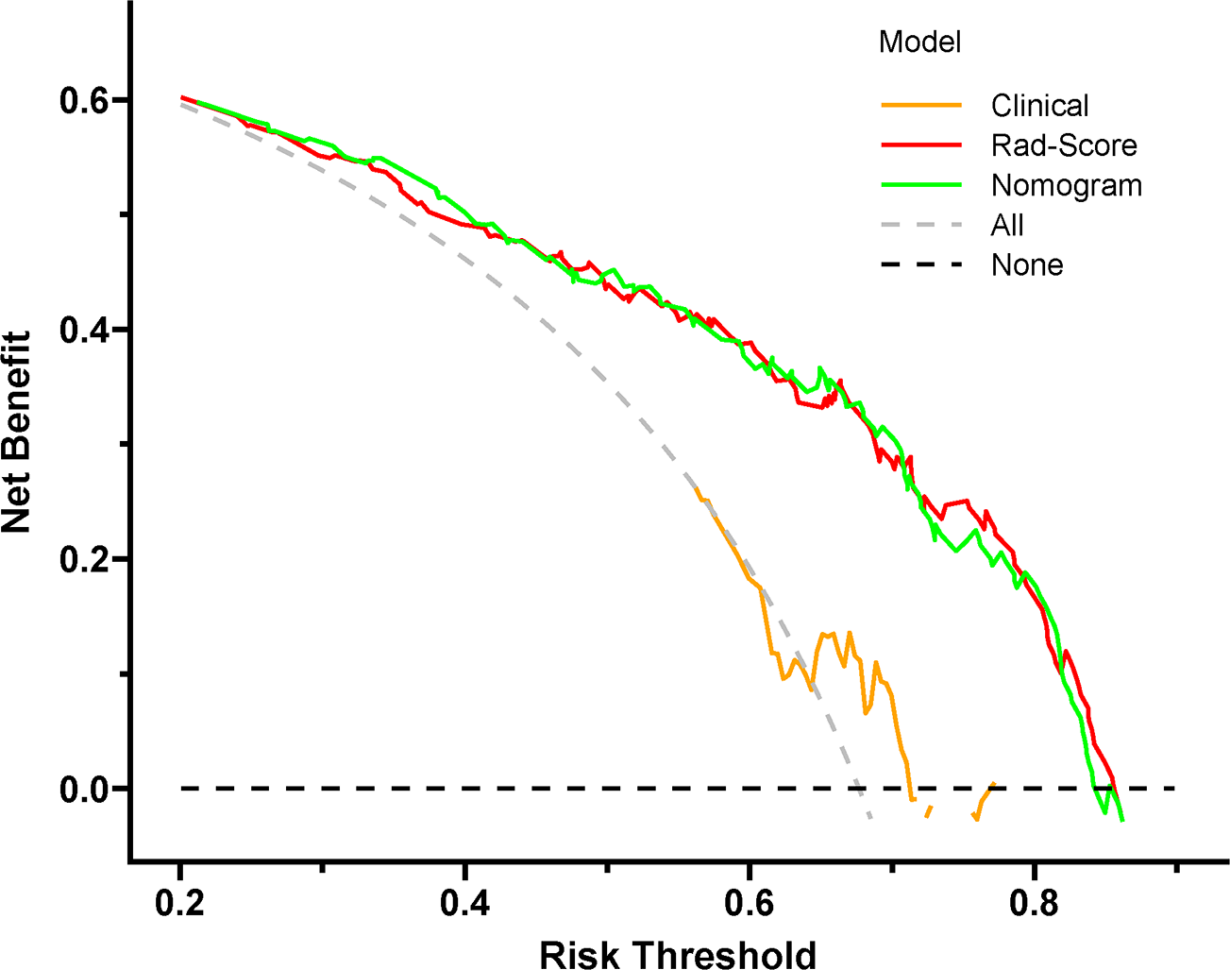
Decision curve of the nomogram model. If the risk threshold is less than 83.8%, the model will obtain more benefit than all treatment (assuming all breast cancer patients were high Ki-67 status) or no treatment (assuming all breast cancer patients were low Ki-67 status).

Decision curve analysis of the clinical, Rad-Score and nomogram models was utilized to select the model that maximized patient benefits. The grey line represents the assumption that all lesions were high Ki-67 status. The black line represents the assumption that all lesions were low Ki-67 status. If the threshold probability was less than 83.8%, using the nomogram model added more benefit (green line) (**Figure 13**).

## Discussion

A number of studies have demonstrated that the Ki-67 index is regarded as one of the most reliable indicator to assess the degree of proliferation of carcinoma cells and is a significant predictive and prognostic factor for patients with BC. Breast carcinoma with high Ki-67 expression responds better to radiotherapy and chemotherapy but is associated with worse prognosis. A meta-analysis (24) including 85 studies found that higher Ki-67 expression was significantly related to a greater risk of recurrence. In addition, Petrelli et al. (25) performed a large meta-analysis including 41 studies and found that there was a significant correlation between the Ki-67 expression and disease-free survival and overall survival. Furthermore, a study by Dowsett and colleagues (26) revealed that the prediction performance of the relapse-free survival could be improved by measuring the Ki-67 index in BC patients receiving short-term endocrine therapy. Therefore, early identification of the Ki-67 status of BC has great significance in aspects of patients’ diagnosis, treatment and prognosis.

In the present study, we studied whether radiomics features extracted from gray-scale ultrasound images of patients with BC could be utilized as a preoperative predictor of the Ki-67 status and proposed a new method to predict the Ki-67 status in patients with BC. A total of 788 ultrasound radiomics features were extracted from each patient with BC. After dimensionality reduction analysis by using the independent sample t test and LASSO regression, we screened out 15 ultrasound radiomics features as imaging markers, and not only established but also validated six advanced machine learning classifiers (DT, RF, SVM, LR, NB and XGBoost) for identifying the Ki-67 status of BC, with AUC values ranging from 0.679 to 1.000 and 0.615 to 0.798 in the training and test sets, respectively. Among them, the LR classifier performed the best in the test set, with the highest AUC value of 0.786, and was obtained as the Rad-Score model. By using the multivariate logistic regression analysis, the age was screened out as a risk factor associated with the high Ki-67 expression. The nomogram model combining the age with Rad-Score was developed and revealed a slightly higher predictive performance than that of Rad-Score model (AUC, 0.808 vs. 0.798) in the test set, and comparative (AUC, 0.790 vs. 0.793) in the training set, revealing that, although Rad-Score had a significant weight in this model, the risk factor of age also had certain value to the predictive performance of the nomogram model in the prediction of the Ki-67 status. Therefore, in this study, the results demonstrated that the Rad-Score model had a high predictive performance for the Ki-67 status in patients with BC, and the nomogram model integrated with the risk factor of age could improve the predictive performance.

The consistency between the model-predicted probability of the Ki-67 status and actual result was evaluated by the calibration curve. The nomogram model showed a good calibration performance with the nonsignificant Hosmer–Lemeshow test statistic in the training and test sets. Compared with the treat-none or treat-all scheme, patients with BC could obtain a significant net benefit from the Rad-Score and nomogram models, which is revealed in decision curve analysis, indicating that both models are valuable in predicting the Ki-67 status. Furthermore, the LGOCV method was performed to verify the reliability and stability of the nomogram model, which yielded a median AUC value of 0.793 in the test set, indicating that the predictive performance of the nomogram model was reliable and robust.

In recent years, a number of studies have demonstrated that radiomics is regarded as an useful and noninvasive method for predicting the Ki-67 status in patients with BC, however, most of the studies are on the basis of mammography and MRI imaging (16, 17, 27–29). Li and colleagues (27) have used radiomics features of intratumoral and peritumoral regions based on breast dynamic contrast-enhanced MRI to identify the HER2 and Ki-67 status, and they reported the combined radiomics signature yielded an AUC of 0.749 for predicting the Ki-67 status in the validation set. Another prior study by Zhang et al. (16) including a total of 128 patients, developing a radiomics model for predicting the Ki-67 proliferation index in patients with invasive ductal breast carcinoma through MRI preoperatively, found that good identification ability was exhibited by the model, with an AUC value of 0.72 in the test set. In contrast, in the present study, the AUC value of the nomogram model was more satisfactory than these reported above in the test set (AUC, 0.808 vs. 0.749 vs. 0.72). In addition, compared with MRI, ultrasound considered as a radiation-free nature, convenient, and reasonable price technology is universally used for breast tumor screening and diagnosis (30, 31). Due to the relatively high predictive performance, it is considered that the nomogram model could be used as a noninvasive and reliable tool in predicting Ki-67 status and assist clinicians for preoperative decision-making.

In our study, 15 key radiomics features were selected to build the Rad-Score model, among which 1 GLDM feature, 4 GLRLM features and 2 GLSZM features were included. These features represent the texture complexity of tumors, which are important in recognizing and classifying internal spatial heterogeneity of the tumor lesions (32, 33), illustrating the importance of texture features in the prediction of high Ki-67 expression. If we can associate the patient’s internal pathways and prognosis with the different texture characteristics of the tumor, it will be useful for the diagnosis and treatment of the patient in the future. In our study, the first-order statistics features such as Skewness, Minimum, Median, RobustMeanAbsoluteDeviation and RootMeanSquared appeared in a high proportion of the final included features, which describe the intensity values of the tumor and are applied to many classification tasks (29, 34). Therefore, radiomics features extracted from ultrasound image of BC could be a potential auxiliary method for clinicians to identify the Ki-67 status.

Wu and colleagues (14) reported that the ultrasound-based radiomics model was an important predictor for the Ki-67 status in patients with ductal carcinoma *in situ* (DCIS). The radiomics signature, which consisted of 51 selected Ki-67 status–related features, achieved perfect predictive efficacy, with AUC values of 0.95 and 0.86 in the training and test sets, which were better than that of the nomogram model in our study (AUC, 0.808 and 0.790 in the training and test sets). However, in their study, only patients with mass type of DCIS were enrolled and the sample size of their retrospective study was smaller (116 vs. 284). In this study, tumors such as invasive ductal carcinoma, invasive lobular carcinoma, as well as mucinous BC were included, which expanded the range of the tumor types. Moreover, compared with Wu et al.’s study, a major highlight of our study was the larger sample size and much more tumor types, which might increase the generalization of the prediction model.

Despite some promising findings, the limitations in our study should be taken into account. First, the statistical power of our retrospective study was limited because of the relatively small sample size. The prediction models were developed and validated for identifying the Ki-67 status with only 284 patients in a single hospital. Therefore, future prospective studies with a larger patient population should be performed to generalize the findings of this study. Second, when the sonographer depicted the ROI manually, there was a certain degree of subjectivity to the contour of the tumor, which might result in poor robustness of the models. However, the evaluation of ICC was performed, and the interobserver reproducibility was well. Third, our radiomics study only used gray-scale ultrasound images, and multi-modal ultrasound such as elastography (35) and contrast-enhanced ultrasound (36) might be taken into account to improve the predictive performance in the future. Forth, only two dimensional analysis of the largest plane of the tumor was applied in our study, which might not comprehensively capture the heterogeneous features of BC. In the future, studies should be carried out to explore the predictive performance of three dimensional analysis for predicting the Ki-67 status in patients with BC. Finally, in this study, the extraction of ultrasound radiomics features required time-consuming tumor contour delineation and artificially predefined features. We believe that deep learning algorithm such as convolutional neural networks (37), which is performed entirely by the machine itself, might accurately and automatically detect and segment and achieve better results.

## Conclusions

In this paper, we proposed a nomogram model based on the clinical risk factor of age and Rad-Score for the preoperative prediction of breast tumor Ki-67 status, and this model showed a high predictive value for the Ki-67 status. This nomogram model is expected to inform treatment strategies and assist clinical decision-making for a personalized treatment in patients with BC. However, further studies with a prospective design and larger population are required to validate the conclusions.

## Data availability statement

The original contributions presented in the study are included in the article/**Supplementary Material**. Further inquiries can be directed to the corresponding authors.

## Ethics statement 

Written informed consent was obtained from the individual(s) for the publication of any potentially identifiable images or data included in this article.

## Author contributions

JW, QF and JY collected the clinical and radiomics data. JW and LG preprocessed patients’ ultrasound imaging and drew the ROI. JW and QF analyzed the data and developed the prediction model. JW wrote the manuscript. ZW, GJ, LH and LG designed the study. All authors contributed to the article and approved the submitted version.

## Funding

This work was supported by Jinhua Science and Technology Bureau Scientific Research Project (2022–3–019).

## Conflict of interest

The authors declare that the research was conducted in the absence of any commercial or financial relationships that could be construed as a potential conflict of interest.

## Publisher’s note

All claims expressed in this article are solely those of the authors and do not necessarily represent those of their affiliated organizations, or those of the publisher, the editors and the reviewers. Any product that may be evaluated in this article, or claim that may be made by its manufacturer, is not guaranteed or endorsed by the publisher.

## Supplementary material

Supplementary Figure 1 Distribution of the 200 AUC values calculated by LGOCV algorithm in the test set.

## Abbreviations

BC, breast carcinoma; Rad-Score, radiomics score; IHC, immunohistochemistry; ADC, apparent diffusion coefficient; DBT, digital breast tomosynthesis; MRI, magnetic resonance imaging; CI, confidence interval; ER, estrogen receptor; PR, progesterone receptor; HER2, human epidermal growth factor receptor 2; FISH, fluorescence *in situ* hybridization; ROI, region of interest; ICC, interclass correlation coefficient; LASSO, least absolute shrinkage and selection operator; DCA, decision analysis curve; ROC, receiver operator characteristic; AUC, area under the curve; SD, standard deviation; GLCM, gray-level co-occurrence matrix; GLRLM, gray-level run length matrix; GLSZM, gray-level size zone matrix; GLDM, gray-level dependence matrix; LR, logistic regression; DT, decision tree; RF, random forest; SVM, support vector machine; NB, naive Bayes; XGB, XGBoost; TP, true positive; FP, false positive; FN, false negative; TN, true negative.
