# Supplementary figures and images for: Integration of ultrasound radiomics features and clinical factors: A nomogram model for identifying the Ki-67 status in patients with breast carcinoma
Source: Front Oncol. 2022 Oct 5;12:979358. doi: 10.3389/fonc.2022.979358 (PMC9581085; doi:10.3389/fonc.2022.979358)

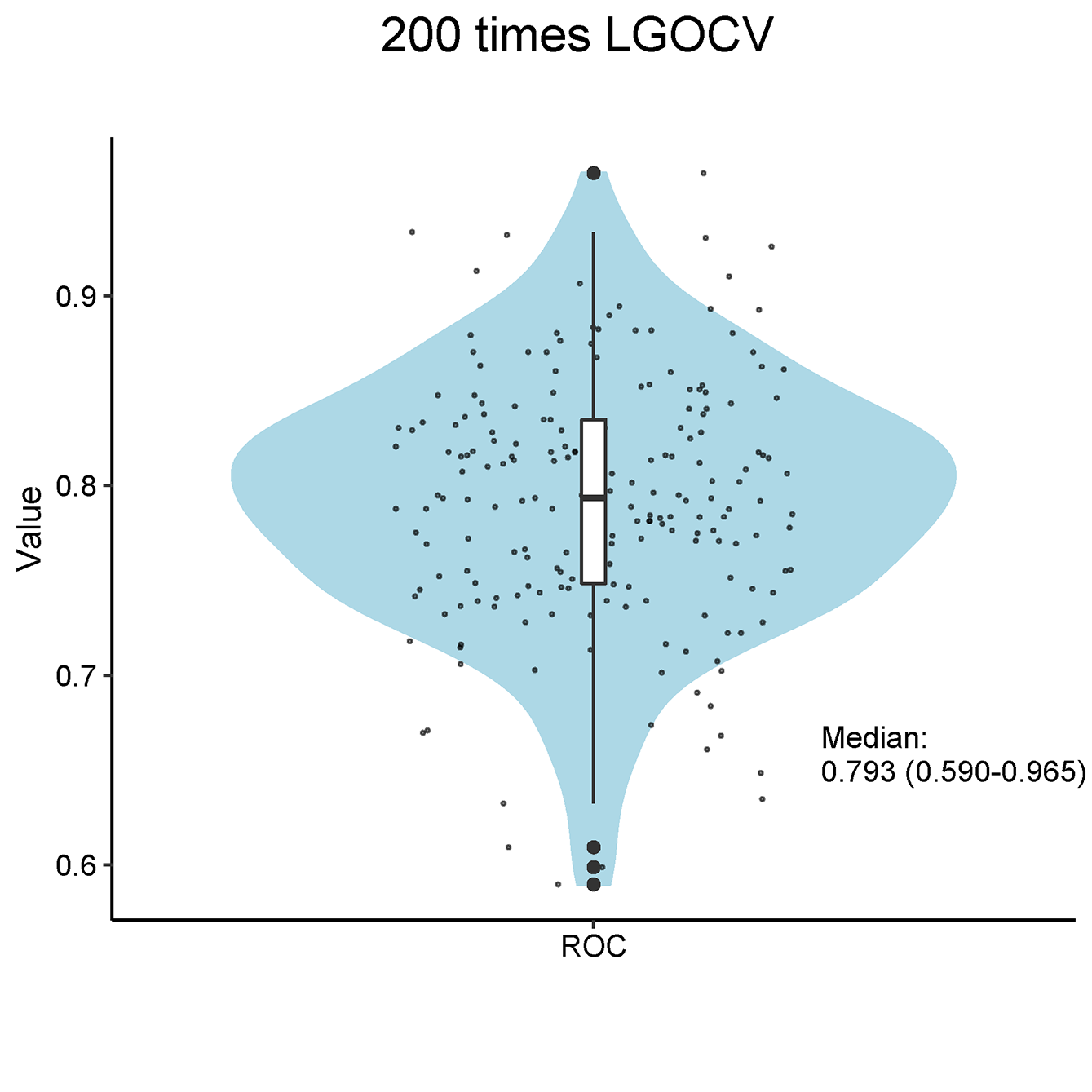

Supplement: Supplementary Figure 1 — Distribution of the 200 AUC values calculated by LGOCV algorithm in the test set. [file Image_1.tif]
