# Supplementary material for: Integration of ultrasound radiomics features and clinical factors: A nomogram model for identifying the Ki-67 status in patients with breast carcinoma
Source: Front Oncol. 2022 Oct 5;12:979358. doi: 10.3389/fonc.2022.979358 (PMC9581085; doi:10.3389/fonc.2022.979358)
Supplement: Supplementary file 2 [file DataSheet_1.docx]

**Rad-score** =

9.865e-01+

2.636e-01*original_shape_MinorAxisLength+ 3.797e-01*original_firstorder_Skewness+ -1.048e-01*original_glszm_SmallAreaEmphasis +

-4.558e-01*wavelet-LLH_firstorder_Minimum+ 1.030e-08*wavelet-LLH_glrlm_HighGrayLevelRunEmphasis+ 1.030e-08*wavelet-LLH_glrlm_LowGrayLevelRunEmphasis+ -4.053e-01*wavelet-LLH_glrlm_RunLengthNonUniformityNormalized+ 8.901e-02*wavelet-LLH_gldm_LargeDependenceLowGrayLevelEmphasis+ 7.471e-01*wavelet-LHL_glrlm_LongRunHighGrayLevelEmphasis+ 2.753e-01*wavelet-LHH_firstorder_Skewness+ 8.198e-01*wavelet-HLL_firstorder_Median+ -6.684e-02*wavelet-HLL_firstorder_RobustMeanAbsoluteDeviation+ -3.479e-01*wavelet-HHL_firstorder_Median+ -2.383e-01*wavelet-HHH_firstorder_RootMeanSquared+ 4.631e-01*wavelet-LLL_glszm_LargeAreaHighGrayLevelEmphasis
