# Supplementary material for: Integration of ultrasound radiomics features and clinical factors: A nomogram model for identifying the Ki-67 status in patients with breast carcinoma
Source: Front Oncol. 2022 Oct 5;12:979358. doi: 10.3389/fonc.2022.979358 (PMC9581085; doi:10.3389/fonc.2022.979358)
Supplement: Supplementary file 3 [file Table_1.docx]

| **Characteristic** | **Total**  **(n=284)** | **High** **Ki-67**  **(n = 196)** | **Low Ki-67**  **(n = 88)** | ***p*-value** |
| --- | --- | --- | --- | --- |
| **Age** (year, mean±SD) | 53.65±10.83 | 52.61±10.65 | 55.95±10.94 | 0.018 |
| **Size** (mm, mean±SD) | 25.09±11.21 | 27.21±10.98 | 20.36±10.30 | <0.001 |
| **Location of disease** |  |  |  | 1.000 |
| Right lobe | 150 | 104 | 46 |  |
| Left lobe | 134 | 92 | 42 |  |
| **ER** |  |  |  | <0.001 |
| Positive | 203 | 123 | 80 |  |
| Negative | 81 | 73 | 8 |  |
| **PR** |  |  |  | <0.001 |
| Positive | 167 | 101 | 66 |  |
| Negative | 117 | 95 | 22 |  |
| **HER2** |  |  |  | <0.001 |
| Positive | 73 | 69 | 4 |  |
| Negative | 211 | 127 | 84 |  |
| **Histologic type** |  |  |  | 0.711 |
| Invasive ductal | 228 | 159 | 69 |  |
| Other | 56 | 37 | 19 |  |
| **Ultrasound equipment** |  |  |  | 1.000 |
| Siemens Acuson S2000 | 233 | 161 | 72 |  |
| LOGIQ E9 | 51 | 35 | 16 |  |
| **US-reported LN** |  |  |  | 0.146 |
| Metastasis positive | 123 | 91 | 32 |  |
| Metastasis negative | 161 | 105 | 56 |  |
| **Pathology-reported LN** |  |  |  | 0.292 |
| Metastasis positive | 160 | 115 | 45 |  |
| Metastasis negative | 124 | 81 | 43 |  |
| **Ki-67** (%, mean±SD) | 29.39±22.96 | 39.31±21.00 | 7.31±3.38 | <0.001 |

**SUPPLEMENTARY TABLE 1 |** Characteristics of patients in the high and low Ki-67 groups.

ER, estrogen receptor; PR, progesterone receptor; HER2, human epidermal growth factor receptor 2; SD, standard deviation; LN, lymph node; US, ultrasound.
