# Supplementary material for: Integration of ultrasound radiomics features and clinical factors: A nomogram model for identifying the Ki-67 status in patients with breast carcinoma
Source: Front Oncol. 2022 Oct 5;12:979358. doi: 10.3389/fonc.2022.979358 (PMC9581085; doi:10.3389/fonc.2022.979358)
Supplement: Supplementary file 4 [file Table_2.docx]

SUPPLEMENTARY TABLE 2 | P values of comparison of AUCs between any pair of the classifiers calculated by DeLong method in the test set

| Model (AUC value) | LR (0.798) | RF (0.756) | SVM (0.726) | XGBoost (0.615) | NB (0.735) | DT (0.638) |
| --- | --- | --- | --- | --- | --- | --- |
| LR (0.798) | 1 | - | - | - | - | - |
| RF (0.756) | 0.355 | 1 | - | - | - | - |
| SVM (0.726) | 0.374 | 0.714 | 1 | - | - | - |
| XGBoost (0.615) | **0.027** | 0.092 | 0.153 | 1 | - | - |
| NB (0.735) | 0.059 | 0.640 | 0.907 | 0.116 | 1 | - |
| DT (0.638) | **0.014** | **0.049** | 0.277 | 0.772 | 0.094 | 1 |

DT, decision tree; RF, random forest; SVM, support vector machine; LR, logistic regression; NB, naive bayes; AUC, area under the curve.
